# Molecular Basis of Ubiquitination Catalyzed by the Bacterial Transglutaminase MavC

**DOI:** 10.1002/advs.202000871

**Published:** 2020-04-30

**Authors:** Hongxin Guan, Jiaqi Fu, Ting Yu, Zhao‐Xi Wang, Ninghai Gan, Yini Huang, Vanja Perčulija, Yu Li, Zhao‐Qing Luo, Songying Ouyang

**Affiliations:** ^1^ The Key Laboratory of Innate Immune Biology of Fujian Province Provincial University Key Laboratory of Cellular Stress Response and Metabolic Regulation Biomedical Research Center of South China Key Laboratory of OptoElectronic Science and Technology for Medicine of the Ministry of Education College of Life Sciences Fujian Normal University Fuzhou 350117 China; ^2^ Laboratory for Marine Biology and Biotechnology Pilot National Laboratory for Marine Science and Technology (Qingdao) Qingdao 266237 China; ^3^ Purdue Institute for Inflammation Immunology and Infectious Disease and Department of Biological Sciences Purdue University West Lafayette IN 47907 USA

**Keywords:** deamidase, effectors, *Legionella pneumophila*, NF‐κB, transglutamination

## Abstract

The *Legionella pneumophila* effector MavC is a transglutaminase that carries out atypical ubiquitination of the host ubiquitin (Ub)‐conjugation enzyme UBE2N by catalyzing the formation of an isopeptide bond between Gln40_Ub_ and Lys92_UBE2N_, which leads to inhibition of signaling in the NF‐κB pathway. In the absence of UBE2N, MavC deamidates Ub at Gln40 or catalyzes self‐ubiquitination. However, the mechanisms underlying these enzymatic activities of MavC are poorly understood at the molecular level. This study reports the structure of the MavC–UBE2N–Ub ternary complex representing a snapshot of MavC‐catalyzed crosslinking of UBE2N and Ub, which reveals the way by which UBE2N–Ub binds to the Insertion and Tail domains of MavC. Based on the structural and experimental data, the catalytic mechanism for the deamidase and transglutaminase activities of MavC is proposed. Finally, by comparing the structures of MavC and MvcA, the homologous protein that reverses MavC‐induced UBE2N ubiquitination, several essential regions and two key residues (Trp255_MavC_ and Phe268_MvcA_) responsible for their respective enzymatic activities are identified. The results provide insights into the mechanisms for substrate recognition and ubiquitination mediated by MavC as well as explanations for the opposite activity of MavC and MvcA in terms of regulation of UBE2N ubiquitination.

## Graphical abstract

The pathogen *Legionella pneumophila* evades immune detection by deploying the transglutaminase effector MavC to ubiquitinate the host ubiquitin‐conjugating enzyme UBE2N. This work presents the structure of MavC–UBE2N–Ub ternary complex and sheds light on the molecular basis for MavC‐induced transglutamination and its mechanistic differences with its homolog MvcA, which counteracts MavC activity by deubiquitinating modified UBE2N in later phases of infection.

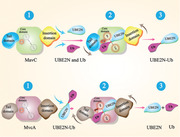

## 1 Introduction

Signal transduction in cells is often mediated by posttranslational modifications (PTMs), which impact the activity of existing proteins to allow rapid responses to upstream cues. Among more than 200 different types of PTMs identified so far,^[^ ^1^ ^]^ ubiquitination is one of the most widely used. Canonical ubiquitination requires the activities of the E1, E2, and E3 enzymes that respectively activate, conjugate, and ligate the 76‐residue ubiquitin (Ub) to substrate proteins.^[^ ^2^ ^]^ Ubiquitination itself is further regulated by ubiquitination and other types of PTMs such as phosphorylation, acetylation, and adenosine diphosphate (ADP)‐ribosylation that target Ub, components of the ubiquitination machinery, or both.^[^ ^3^ ^]^ This complex crosstalk among various PTMs allows cells to achieve better fine‐tuning of their response to various stimuli, particularly under disease conditions.^[^ ^3^, ^4^ ^]^

Pathogens have evolved diverse mechanisms to co‐opt host functions to promote their fitness. One such mechanism is the acquisition of virulence factors capable of effective modulation of cellular processes by various PTMs.^[^ ^5^ ^]^ *Legionella pneumophila*, the causative agent of Legionnaires’ disease, is one such example. The intracellular life cycle of this bacterium utilizes the Dot/Icm type IV secretion system that injects hundreds of virulence factors known as effectors into host cells.^[^ ^6^ ^]^ These effectors extensively modulate cell signaling hubs such as small GTPases and the Ub network to create a niche permissive for intracellular replication of the *L. pneumophila*.^[^ ^7^ ^]^

Co‐option of the host Ub network by *L. pneumophila* appears to be of particular importance for modulating host cellular immune process to facilitate its intracellular replication. More than 10 effectors with E3 Ub ligase activity have been identified. Although their target proteins remain elusive in most cases, these effectors cooperate with E1 and E2 enzymes in host cells to form active ubiquitination machineries (Figure S1A, Supporting Information).^[^ ^8^ ^]^ A paradigm shift discovery was made by the study of the SidE effector family (SidEs) that includes effectors such as SdeA, which catalyze NAD^+^‐dependent ubiquitination. This mechanism involves Ub activation via ADP‐ribosylation and phosphodiesterase (PDE)‐mediated ligation of phosphoribosylated ubiquitin (PR‐Ub) onto serine residues of substrate proteins (Figure S1B, Supporting Information).^[^ ^9^ ^]^ Interestingly, two research groups recently reported that DupA and DupB, the two highly homologous PDE domain‐containing deubiquitinases from *L. pneumophila*, similarly reverse phosphoribosyl serine ubiquitination on their substrates (Figure S1B, Supporting Information).^[^ ^10^ ^]^ Moreover, the activity of SidEs is regulated by SidJ, another effector which inhibits the mono‐ADP‐ribosyltransferase activity by calmodulin‐dependent glutamylation (Figure S1B, Supporting Information).^[^ ^11^ ^]^

The modification of the E2 enzyme UBE2N by MavC represents another atypical ubiquitination mechanism. In this reaction, UBE2N is ligated to Ub via an isopeptide bond formed between Gln40 of Ub and Lys92 (i.e., γ‐glutamyl‐ε‐Lys bond between Ub_Gln40_ and UBE2N_Lys92_) or, to a lesser extent, Lys94 of UBE2N.^[11c]^ This ligation is mediated by transglutamination, a reaction that does not require exogenous energy.^[^ ^12^ ^]^ Analogously to other transglutaminases that function as deamidases in the absence of their target substrates.^[^ ^13^ ^]^ MavC uses catalytic Cys74 that is crucial for both enzymatic activities (Figure S1C,D, Supporting Information).^[^ ^14^ ^]^ Ubiquitination at Lys92 abolishes the activity of UBE2N, which in turn curbs the formation of K63‐type polyubiquitin chains through canonical ubiquitination otherwise mediated by UBE2N, E1 and UVE1, thereby inhibiting NF‐κB activation (Figure S1C, Supporting Information).^[11c]^

**Figure 1:**
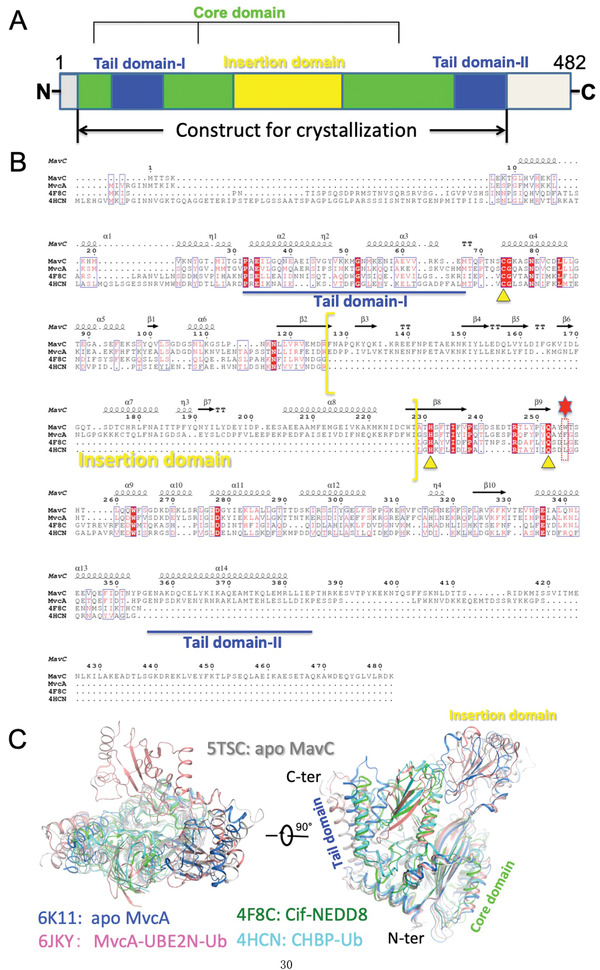
Primary sequence and 3D structure comparisons of MavC and its homologs MvcA, Cif and CHBP. A) Domain organization of MavC. The Core domain, Insertion domain, and Tail domain (divided into Tail domain‐I and ‐II) are colored green, yellow, and blue, respectively. B) Primary sequence alignment of MavC with MvcA, Cif, and CHBP generated by ClusterW (https://www.genome.jp/tools-bin/clustalw) and ESpript 3 (http://espript.ibcp.fr/ESPript/ ESPript/). Every tenth residue is indicated with a dot (.) above it. Strictly conserved residues are indicated in white on a red background. The yellow triangles indicate the three residues of catalytic triad sites. Residues Trp255 of MavC and Phe268 of MvcA proximal to the active site are marked by a red dotted rectangle box and a red hexagon above them. The part of sequence corresponding to the Insertion domain is enclosed by yellow brackets, whereas the parts of sequence corresponding to the Tail domain (‐I and ‐II) are underlined by a blue line. C) 3D structure comparison of apo MavC (PDB ID: 5TSC) with MvcA (PDB ID: 6K11 and 6JKY), Cif (PDB ID: 4F8C), and CHBP (PDB ID: 4HCN) in two different orientations.

MavC and its homolog MvcA are structurally similar^[^ ^14^ ^]^ to the canonical ubiquitin deamidase cycle inhibiting factor (Cif) effectors from enteropathogenic *Escherichia coli* and its homolog in *Burkholderia pseudomallei* (CHBP) (**Figure** **1**).^[^ ^15^ ^]^ Both MavC and MvcA have ubiquitin deamidase activity but only MavC is able to induce monoubiquitination of UBE2N. Furthermore, we recently found that MvcA counteracts the transglutamination activity of MavC by removing ubiquitin from UBE2N‐Ub.^[^ ^12^ ^]^ However, although the structures of MavC and its homolog MvcA have been solved (Figure 1C),^[^ ^14^ ^]^ neither the mechanism underlying transglutaminase‐induced UBE2N ubiquitination by MavC nor the molecular basis for their opposite catalytic activities has been elucidated. Here, by solving the structure of the MavC–UBE2N–Ub ternary complex and comparing it to other available structures of MavC and MvcA, we illustrate the structural basis for substrate recognition by MavC and the mechanism that mediates the formation of the isopeptide bond between Lys92 in UBE2N and Gln40 in Ub. In addition, structural comparison of the MavC and MvcA in their apo form and in the ternary complex has allowed us to gain insights into the basis of the opposite biochemical activity exhibited by these two highly similar proteins in terms of regulation of UBE2N ubiquitination.

## 2 Results

### 2.1 The Insertion Domain of MavC is Essential for UBE2N Ubiquitination but Not for Self‐Ubiquitination and Ubiquitin Deamidation Activities of MavC

**Figure 2:**
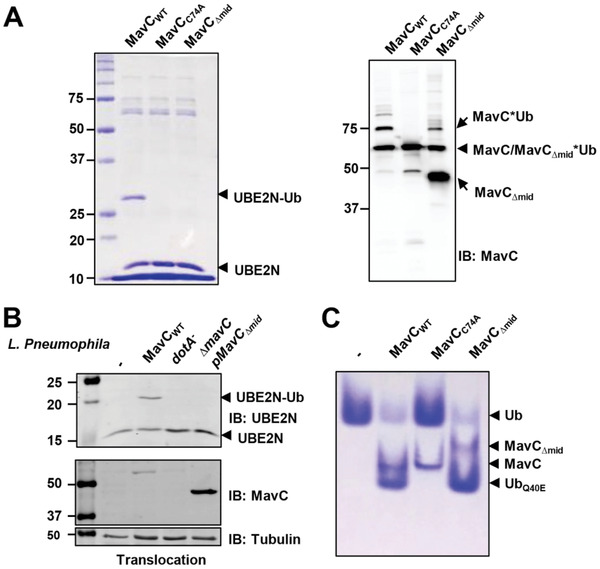
The Insertion domain of MavC is essential for UBE2N ubiquitination but not for ubiquitin deamidation and MavC self‐ubiquitination. A) Deletion of the Insertion domain of MavC (MavC_∆mid_) abolished UBE2N ubiquitination but had no effect on ubiquitin deamidation and MavC self‐ubiquitination. Ubiquitination reactions containing MavC, MavC_C74A_, or MavC_∆mid_ were resolved by SDS‐PAGE and visualized by Coomassie staining (left panel) or immunoblotting with MavC‐specific antibodies (right panel). B) The MavC_∆mid_ mutant did not catalyze UBE2N ubiquitination in cells infected by *L. pneumophila*. U937 cells were infected with the indicated *L. pneumophila* strains for 1 h at a MOI of 10, after which the cell lysates separated by SDS‐PAGE were probed by immunoblotting with the indicated antibodies. Note that the MavC_∆mid_ mutant was translocated into host cells at levels higher than wild‐type bacteria. C) MavC mutant lacking the Insertion domain is capable of ubiquitin deamidation. Proteins in reactions containing ubiquitin and MavC or its mutants were separated by native PAGE and visualized by Coomassie staining.

Purified MavC from *E. coli* exists primarily as a mixture of monomers and dimers in solution, both of which interact with UBE2N in size‐exclusion chromatography (SEC) (Figure S2, Supporting Information). Unlike Cif and CHBP, both apo MavC (PDB ID: 5TSC) and MvcA (PDB ID: 6K11) possess a unique Insertion domain, which likely plays an important role in interaction with UBE2N (Figure 1B,C).^[^ ^14^ ^]^ To test this hypothesis, we constructed a MavC truncation mutant missing Insertion domain (MavC_∆mid_, lacks residues Gln131 to Asn223) and then examined its activities (Figure 1A). In contrast to wild‐type MavC (MavC_WT_) that robustly induced UBE2N ubiquitination, both MavC_C74A_ and MavC_∆mid_ have lost the ability to carry out UBE2N ubiquitination (**Figure** **2**A, left panel). In line with the biochemical results, although MavC_∆mid_ displayed high expression levels in the *L. pneumophila* strain *∆mavC* and was delivered into host cells, it failed to ubiquitinate UBE2N (Figure 2B). Intriguingly, MavC_∆mid_ is still capable of catalyzing self‐ubiquitination (Figure 2A, right panel) and ubiquitin deamidation (Figure 2C). These results suggest that the Insertion domain is essential for UBE2N ubiquitination but not for ubiquitin deamidation and self‐ubiquitination activities of MavC. Considering these findings, we further hypothesized that the Insertion domain of MavC is involved in substrate recognition.

### 2.2 Binding Affinities between MavC and Its Substrates

We first examined the binding affinity between Ub and MavC. Although Ub participates in MavC‐induced transglutamination in vitro, we could not observe the formation of a stable complex between Ub and MavC_C74A_ in solution using SEC and no detectable binding was observed in MST assays (Figure S3A, Supporting Information). High concentrations of Ub (>3 × 10^−3^ m) were insufficient to generate saturation curve in MST analysis. We therefore assumed that binding between these two proteins is too weak to be detected under the assay conditions. Similarly, we did not observe crystal growth when mixing MavC and Ub at 1:3 ratio. These findings are inconsistent with those of a previous study that confirmed binding between MavC and Ub by monitoring chemical shift perturbations (CSPs) in NMR titration experiments.^[^ ^14^ ^]^ The differences are probably due to the fact that the catalytically active MavC_WT_ used in this previous study deamidated the ^15^N‐labeled ubiquitin during titration in solution nuclear magnetic resonance (NMR).^[^ ^14^ ^]^ Thus, although Ub participates in MavC‐mediated transglutamination and deamidation reactions, the strength of their interactions varies when assayed with enzymatically active or inactive MavC.^[^ ^12^ ^]^

We also examined binding between MavC_C74A_ and UBE2N with the MST assay and found that these two molecules bind at a *K*_d_ value of 1.14 × 10^−6^ m in solution (Figure S3B, Supporting Information). Moreover, since the best resolution we could obtain for the MavC_C74A_–UBE2N binary complex was only about 3.5 Å, we speculated that the intermolecular interactions mediated by the Insertion domain of MavC are dynamic. Interestingly, although both MavC and MvcA contain an Insertion domain, MvcA has been shown to have no interaction with nonubiquitinated UBE2N.^[^ ^14^ ^]^ Taken together, these results indicate that, in spite of ≈50% sequence identity, MavC and MvcA differ in UBE2N recognition.

To investigate the molecular basis for substrate binding and transglutaminase activity of MavC, we aimed to solve the structure of the MavC–UBE2N–Ub ternary complex. We attempted to improve the binding stability between MavC_C74A_ and its substrates Ub and UBE2N by directly supplying the product of MavC‐catalyzed transglutamination on the premise that the binding affinity between MavC and crosslinked UBE2N–Ub binary complex (*K*_d_ = 1.4 × 10^−6^ m) is similar to the binding affinity between MavC and UBE2N, which would thus circumvent the problem of weak interactions between and MavC and free Ub (Figure S3C, Supporting Information). The MavC_C74A_ and UBE2N_K94A_ mutants were used for crystallization experiments to ensure that UBE2N is ubiquitinated only at Lys92.^[^ ^12^ ^]^

### 2.3 Overall Structure of MavC in Complex with its Product UBE2N–Ub and Catalytic Site Interactions

**Figure 3:**
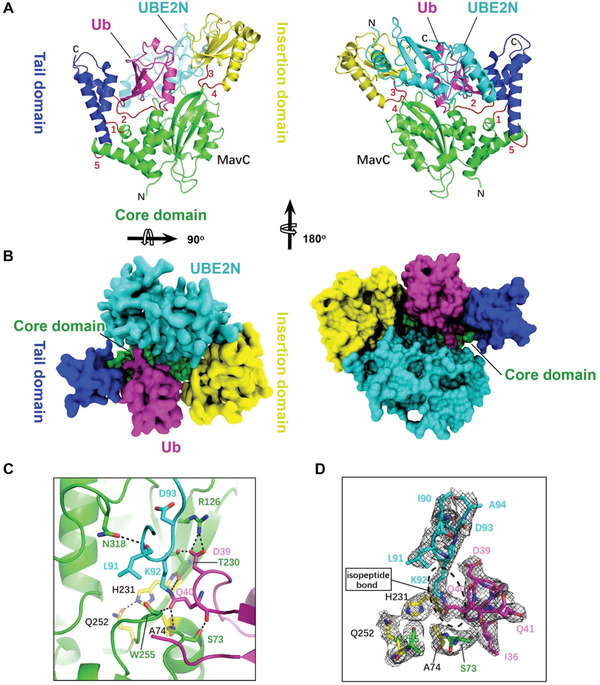
The overall structure of the MavC–UBE2N–Ub complex. A) Ribbon representation of the MavC–UBE2N–Ub ternary complex. In MavC, the Tail domain (helices α2, α3, and α14, blue) is linked to the Core domain (green) by three loops (loops 1, 2, and 5, red), whereas the Insertion domain (yellow) is linked to the Core domain by two loops (loops 3 and 4, red). Ub and UBE2N are shown in magenta and cyan, respectively. The view in right panel is generated by rotating the image in left panel by 180^o^ around the indicated axis. B) Top view of the MavC–UBE2N–Ub ternary complex with surface representation. The color of the Tail domain, Core domain, Insertion domain, Ub, and UBE2N is shown same as (A). The view in right panel is generated by rotating the image in left panel by 180^o^ around the indicated axis. C) MavC‐induced linkage between Lys92 of UBE2N (cyan) and Gln40 of Ub (magenta). The catalytic triad (yellow) of MavC (Cys74 was mutated to Ala in our structure) and other residues participating in the reaction are shown as sticks. Hydrogen bonds are shown as dashed lines. D) The 2Fo‐Fc map of key residues around active site contoured at 1.2 *σ*. The catalytic triad (Ala(Cys)74‐His231‐Gln252, yellow) and Ser73 of MavC, Ile90, Leu91, Lys92, Asp93, and Ala94 of UBE2N, and Ile36, Asp39, Gln40, and Gln41 of Ub are shown as sticks.

By following the protocol described above, we successfully crystallized and solved the structure of the MavC_C74A_–UBE2N_K94A_–Ub ternary complex at a 2.85 Å resolution (**Table** **1**). Only one copy of the ternary complex could be found in the asymmetric unit (ASU). Interestingly, the architecture of the MavC_C74A_–UBE2N_K94A_–Ub complex is highly similar to that of the MvcA–UBE2N–Ub complex reported in our previous study.^[^ ^12^ ^]^ The ternary complex solved in this study represents the stage of MavC‐mediated UBE2N ubiquitination in which UBE2N and Ub have already been crosslinked by an isopeptide bond but are still bound to MavC (**Figure** **3**A,B; Movie S1, Supporting Information). MavC, which assumes a concave shape, consists of a Core domain flanked by a helical Tail domain and an Insertion domain (Figures 1 and 3A,B). Loops 1, 2, and 5 along with loops 3 and 4 serve as flexible hinges that respectively link the Tail domain and the Insertion domain to the Core domain, thus conferring the flexibility to the two subdomains required for binding UBE2N–Ub (Figure 3A,B). The covalently linked UBE2N–Ub sits in the groove formed by the Tail domain and the Insertion domain of MavC with the UBE2N and Ub portion of the molecule hanging on either side of the enzyme.

**Table 1:** X‐ray crystallography data collection and refinement statistics

Dataset	MavC_C74A_–UBE2N_K92A_–Ub
Data collection	
Beamline	BL‐17U1, SSRF
Wavelength [Å]	0.9792
Resolution range^a^^,^ b ^)^	48.96–2.85 (2.90–2.85)
Space group	*P*6_5_
Cell dimensions	
*a*, *b*, *c* [Å]	149.56, 149.56, 58.77
*α*, *β*, *γ* [°]	90.00, 90.00, 120.00
Total reflections	2130237
Unique reflections	17794 (882)
Multiplicity	20.0
Completeness [%]	99.94 (99.89)
Mean *I*/sigma (*I*)	35.5 (2.45)
*R*‐merge	0.103 (1.275)
*R*‐meas	0.106 (1.308)
*R*‐pim	0.024 (0.291)
CC1/2	1.003 (0.940)
Refinement	
Reflections used in refinement	17773 (1763)
Reflections used for *R*‐free	824 (66)
*R*_work_	0.194 (0.354)
*R*_free_	0.246 (0.460)
Wilson *B*‐factor [Å^2^]	76.60
Number of nonhydrogen atoms	4779
Macromolecules	4779
Protein residues	602
RMS (bonds)	0.012
RMS (angles)	1.67
Ramachandran favored [%]	91.11
Ramachandran allowed [%]	8.39
Ramachandran outliers [%]	0.50
Rotamer outliers [%]	10.71
Clashscore	13.00
Average *B*‐factor [Å^2^]	81.0

a) For each structure one crystal was used;

b) Values in parentheses are for highest‐resolution shell.

The catalytic site for transglutamination is situated at the bottom of the concavity of MavC, with the Cys74‐His231‐Gln252 catalytic triad (Cys74 was mutated to Ala in our structure) at the center of the concave line (Figure 3C–D). Several residues adjacent to the catalytic triad contribute to the stabilization of the UBE2N loop and the Ub loop that respectively contain Lys92 and Gln40, the two reactive residues covalently bonded by transglutamination. Ala74, Thr230, and Trp255 of MavC interact with Gln40 of Ub through hydrogen bonding that involves both side chain and backbone atoms. Moreover, Arg126 and Thr230 of MavC form three pairs of hydrogen bonds with Asp39 of Ub, thereby further stabilizing the Gln40‐containing loop. The UBE2N loop containing Lys92 is held in place by hydrogen bonding between Asn318 of MavC and Leu91 of UBE2N (Figure 3C–D).

### 2.4 Key Residues Mediating Interactions between MavC and UBE2N and Their Implications in Transglutaminase Activity of MavC

**Figure 4:**
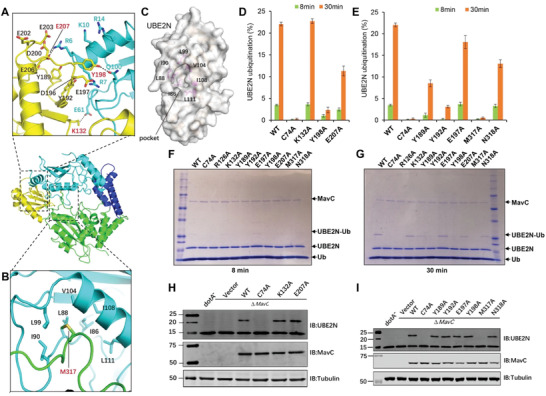
The effects of mutations in MavC on MavC‐induced ubiquitination. A,B) Detailed views of the interactions between MavC and UBE2N in the ternary complex. A) Three pairs of hydrogen bonds formed between the Insertion domain of MavC and UBE2N are shown as dashed lines, and the hydrogen‐bonded residues are labeled with red numbers. Residues involved in electrostatic interactions between the negative surface of MavC and positive surface of UBE2N are labeled with black (MavC) and blue (UBE2N) numbers. The interacting residues are colored yellow (Insertion domain) and cyan (UBE2N). B) The hydrophobic residues forming pocket 2 of UBE2N are shown as cyan sticks, whereas Met317 of MavC that inserts into the pocket is shown as a green stick and labeled by red number (B). C) The hydrophobic pocket of UBE2N (designated pocket 2) into which Met317 of MavC inserts in the ternary complex. The hydrophobic residues lining the pocket (magenta sticks) are labeled with numbers in black and UBE2N is shown as cartoon with partially transparent surface (gray). D,E) Mutational analysis of MavC residues involved in the MavC–UBE2N interaction interface and their importance for UBE2N ubiquitination. MavC or its mutant derivatives were added to reactions containing UBE2N and ubiquitin for 8 or 30 min at 37 °C. Samples resolved by SDS‐PAGE and visualized by Coomassie staining were quantified using Image Studio. The ratios are from three independent experiments. Error bars indicate standard error of the mean (SEM). F,G) SDS‐PAGE images of the ubiquitination assay shown in panels (D) and (E). MavC and its mutant derivatives were added to reactions containing UBE2N and Ub, the reactions were allowed to proceed for 8 or 30 min at 37 °C before SDS‐PAGE and visualization with Coomassie staining. H,I) The ability of MavC mutant derivatives to catalyze UBE2N ubiquitination during *L. pneumophila* infection. Plasmids carrying *mavC* mutants were introduced into the ∆*mavC* mutant and the bacteria were used to infect U937 cells. Saponin‐soluble lysates of infected cells resolved by SDS‐PAGE were probed with antibodies specific for MavC or UBE2N.

In the ternary complex, interactions between MavC and UBE2N include hydrogen bonding, electrostatic and hydrophobic interactions. Three pairs of hydrogen bonds (Lys132‐Glu61, Glu207‐Arg, and Tyr198‐Gln100) at the interface between the Insertion domain of MavC and UBE2N contribute to UBE2N recognition (**Figure** **4**A). Electrostatic interactions further stabilize the binding of UBE2N–Ub with MavC. These electrostatic interactions involve the loop between β6 strand and α7 helix composed of seven negatively charged residues (Asp196, Glu197, Asp200, Glu202, Glu203, Glu206, and Glu207) and three Tyr residues (Tyr189, Tyr192, and Tyr198) that interacts with a positively charged region formed by four residues (Arg6, Arg7, Lys10, and Arg14) of the α1 helix in UBE2N (Figure 4A). According to the sequence conservation analysis, the above residues of MavC are conserved except Tyr189, Glu197, and Glu202 (Figure S4, Supporting Information). Hydrophobic interactions are mediated by Met317 from the Core domain of MavC which inserts into a hydrophobic pocket in UBE2N formed by residues Ile86, Leu88, Ile90, Leu99, Val104, Ile108, and Leu111 (Figure 4B,C).

To determine the impact of the residues that play role in interaction and recognition of UBE2N on ubiquitination, we designed a set of MavC mutants and tested their ability to produce UBE2N–Ub. Mutations of Tyr192, Tyr198, and Met317 to alanine severely impaired ubiquitination of UBE2N, whereas the MavC_Y189A_ and MavC_N318A_ mutants were only partially defective. Furthermore, mutation to alanine of Glu207 that hydrogen bonds with Arg of UBE2N caused a slight defect in catalyzing UBE2N ubiquitination, suggesting that this residue is required for optimal ubiquitination activity. In contrast, we did not detect defects associated with MavC_E197A_ (Figure 4D,E). These observations are consistent with the binding results, which showed that MavC_Y192A_ and MavC_Y198A_ failed to bind UBE2N (Figure 4F,G). When introduced into the *L. pneumophila ∆mavC* mutant on a plasmid, each of the abovementioned mutants can be expressed and translocated into host cells at levels comparable to that of wild‐type MavC. Our in cellular results reveal that only the MavC _M317A_ mutant has lost the ability to induce UBE2N ubiquitination, implying an important role of this residue in the transglutaminase activity of MavC (Figure 4H,I). Therefore, electrostatic interactions and hydrophobic interactions mediated by Met317 are the main force that stabilizes the binding of UBE2N–Ub with MavC.

**Figure 5:**
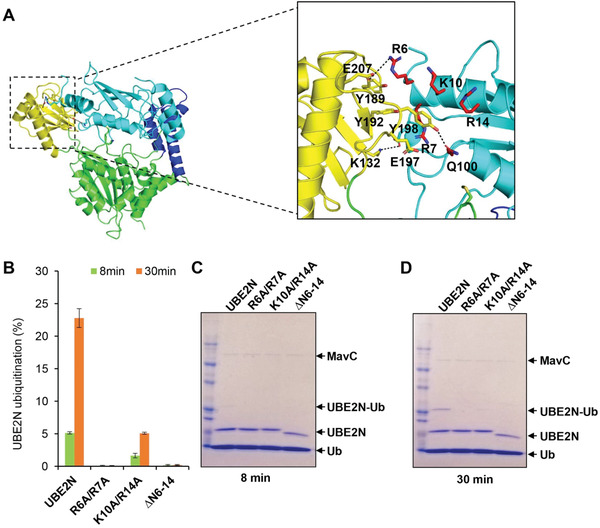
The effects of mutations in UBE2N on MavC‐induced ubiquitination. A) Detailed views of the interactions between MavC and UBE2N in the ternary complex, similar to Figure 4A. B) The effects of mutations in UBE2N on MavC‐induced ubiquitination. MavC was added to reactions containing ubiquitin, UBE2N or UBE2N_R6A/R7A_, UBE2N_K10A/R14A_, and UBE2N_∆N6‐14_ (deletion mutant missing the residues from Arg6 to Arg14) mutants. The reactions were allowed to proceed for 8 or 30 min at 37 °C. Samples resolved by SDS‐PAGE and visualized by Coomassie staining were quantified using Image Studio. The ratios are from three independent experiments. Error bars indicate standard error of the mean (SEM). C,D) The effects of mutations in UBE2N on MavC‐induced ubiquitination. MavC was added to reactions containing ubiquitin, UBE2N or its mutants. The reactions were allowed to proceed for 8 or 30 min at 37 °C before SDS‐PAGE and detection by Coomassie staining.

To further validate the substrate recognition mechanism implied by the ternary structure, we designed UBE2N_R6A/R7A_, UBE2N_K10A/R14A_, and UBE2N_∆N6‐14_ (deletion mutant missing the residues from Arg6 to Arg14) mutants predicted to affect intermolecular electrostatic interactions and tested them for ubiquitination using the method described above. We found that neither UBE2N_R6A/R7A_ nor UBE2N_∆N6‐14_ demonstrated detectable ubiquitination and that UBE2N_K10A/R14A_ can still be ubiquitinated but at a markedly reduced level (**Figure** **5**A–D).

### 2.5 Interactions between MavC and Ub

Although MavC_C74A_ was not found to interact with Ub directly in SEC and does not exhibit observable affinity for it in MST assays, the structure of our ternary complex structure clearly shows that MavC_C74A_ indeed interacts with Ub via five distinct contact regions (termed contact regions A–D and the carboxyl terminus contact region CTC) on the Tail domain of MavC. Several pairs of hydrogen bonds and hydrophobic interactions from these regions contribute to positioning of the ubiquitin molecule optimal for transglutamination (Figure S5A,B, Supporting Information). Contact region A is formed by a hydrogen bond between Glu42 of MavC and Lys6 of ubiquitin (Figure S5C, Supporting Information). Contact region B involves the loop of the N‐terminal β‐hairpin in Ub, particularly residues Leu8, Thr9, and Gly10, which engage in hydrophobic interactions with a hydrophobic pocket of MavC formed by residues Ile31, Pro32, Ile35, Leu36 (Figure S5D, Supporting Information). In contact region C, three pairs of hydrogen bonds are formed, namely Gln31(Ub):Glu123(MavC), Glu34(Ub):Asn79(MavC), and Gly35(Ub):Arg121(MavC) (Figure S5E, Supporting Information). Contact region D features interactions mediated by a single pair of hydrogen bonds formed between Asn25 of ubiquitin and Ile163 of MavC (Figure S5F, Supporting Information). Finally, the flexible tail at the C‐terminus of Ub is held in place by contacting an adjacent groove of MavC through hydrogen‐bonding involving Glu66, Gln69, Thr71, Asn72, Ser73, Ser257, His258 of MavC and Leu71, Arg72, Leu73, Arg74 of the ubiquitin tail (Figure S5G, Supporting Information).

### 2.6 Rotation of the Insertion and Tail Domains of MavC is Essential for Delivering UBE2N and Ub to the Transglutaminase Active Site

**Figure 6:**
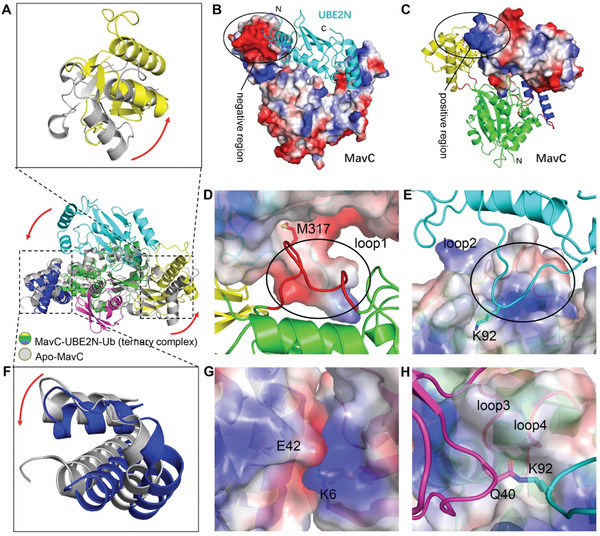
Structural details of UBE2N and Ub recognition by MavC. A) Superimposition of Insertion domains of UBE2N–Ub bound and apo MavC. The rotation of the Insertion domain from its position in apo MavC to its position in the ternary complex is indicated by an arrow. B,C) The Insertion domain of MavC in the ternary complex undergoes a rotation during the catalysis that links UBE2N and Ub. Positively charged residues of UBE2N (cyan cartoon) and the negatively charged region of MavC are indicated by a circle (B). Negatively charged residues of the Insertion domain of MavC–UBE2N–Ub (yellow cartoon) and positively charged region of UBE2N (cyan cartoon) are indicated by a circle (C). D,E) Two loops stabilize the binding of UBE2N to MavC. Loop1 (Leu88‐Trp95, red cartoon) of MavC is indicated by a circle. Met317 of MavC is shown as red stick which inserts into the pocket of UBE2N electrostatic surface (D). Loop2 (His311‐Lys320) of UBE2N is indicated by a circle. Lys92 of UBE2N is shown as cyan stick which inserts into the pocket of ternary complex electrostatic surface (E). F) Superimposition of Tail domains of UBE2N–Ub bound and apo MavC. The rotation of the Tail domain from its position in apo MavC to its position in the ternary complex is indicated by an arrow. G) The interacting interface of MavC–UBE2N–Ub Tail domain and Ub are shown as electrostatic surface. Negatively charged Glu42 and positively charged Lys6 are shown as sticks. H) Two loops stabilize the binding of isopetide bond between UBE2N and Ub to MavC. The isopetide bond between UBE2N (cyan) and Ub (magenta) is shown as sticks. Two hydrophobic loops (loop3 and loop4) are shown as red cartoon and labeled on the outer side of the electrostatic surface.

In comparison to the apo form, the Insertion domain of MavC underwent a distinct counterclockwise rotation of ≈30° in the ternary complex. This movement allowed the formation of electrostatic interactions and hydrogen bonds between the Insertion domain and a positively charged region of UBE2N (**Figure** **6**A–C; Movie S1, Supporting Information). The loop 1 (Leu88‐Trp95) of UBE2N and the loop2 (His311‐Lys320) of MavC interlock and thus strengthen binding between MavC and UBE2N and firmly stabilize the Lys92 of UBE2N in the enzyme activation center (Figure 6D,E). Similarly to Insertion domain, the Tail domain of MavC also rotates counterclockwise relative to its position in apo form MavC. The rotation of the Tail domain instigates hydrogen bonding between Gln42 of MavC and Lys6 of Ub and electrostatic interactions (Figure 6F,G). In addition, the loop 3 (Thr71‐Ser73) and loop 4 (Gln252‐Ser257) play an important role in maintaining the Lys92 of UBE2N and Gln40 of Ub in the precise location of the enzyme activation center (Figure 6H). Based on these findings, we propose that counterclockwise rotation of the Insertion and Tail domains in MavC during ternary complex formation moves Lys92 of UBE2N and Gln40 of Ub closer to the catalytic center, while the Core domain serves as a scaffold to stabilize UBE2N_Lys92_ and Ub_Gln40_ in the catalytic center, so that these three domains complete the transglutamination reaction cooperatively.

### 2.7 The Mechanism for MavC‐Mediated Transglutamination and Molecular Basis of Opposite Enzymatic Activities of MavC and MvcA

MvcA is a deubiquitinase that counteracts the activity of MavC by removing ubiquitin from UBE2N–Ub.^[^ ^12^ ^]^ However, in spite of their opposite enzymatic activities, these two proteins share ≈50% identity and their structures are highly similar (Figure 1B). Apo forms of MavC and MvcA represent the closed catalytically inactive form of the two proteins characterized by high conformational stability, which can be seen when superimposing three available structures of apo MvcA (PDB ID: 5SUJ,^[^ ^14^ ^]^ 5YM9, and also our previous work with PDB ID: 6K11^[^ ^12^ ^]^)—the three structures and all monomers in an asymmetrical unit align with a maximal RMSD of 0.613 Å (Figure S6A, Supporting Information). Hence, we used the apo form structures as a reliable reference point in our attempt to determine functional divergence of MavC and MvcA by comparing the structures of MavC and MvcA in their apo form and in ternary complexes. Interestingly, the structural similarity between MavC and MvcA from the respective ternary complexes is markedly higher than that between apo MavC and apo MvcA (Figure S6B–E, Supporting Information). This suggests that the two proteins undergo significant conformational changes during transition to ternary complexes. In both proteins, these conformational changes can be mainly contributed to rotational movement of the Insertion domain and Tail domain relative to the Core domain.

**Figure 7:**
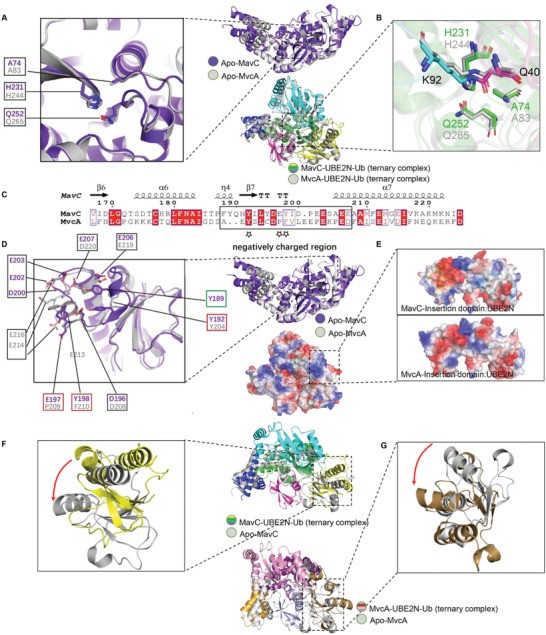
Structural comparison and analysis of Insertion domains and catalytic triads of MavC and MvcA. A,B) Superimposition of the catalytic triads (panel A) of apo MavC (purple) and apo MvcA (gray). Superimposition of the catalytic triads (panel B) of MavC–UBE2N–Ub (colorized) and MavC–UBE2N–Ub (gray) ternary complexes. The catalytic triads of MavC and MvcA are shown as sticks. C) Structure‐based sequence alignment of the loop between β6 and α7 that contains negatively charged amino acids. The negatively charged region is boxed by black rectangle, and the key residues involved in the interactions between MavC and UBE2N are labeled by stars. D,E) Structural comparison of Insertion domains of MavC and MvcA. The residues involved in the formation of the negatively charged region are shown as sticks, corresponding residues in MavC and MvcA are labeled by black boxes, and the key residues are highlighted by red boxes (panel D). Superimposition of the Insertion domains of MavC–UBE2N–Ub and MavC–UBE2N–Ub ternary complexes. The Insertion domains of MavC and MvcA are shown as electrostatic surface (panel E). F,G) Superimposition of Insertion domains of MavC–UBE2N–Ub ternary complex (colorized) and apo MavC (gray) (panel F), and MvcA–UBE2N–Ub ternary complex (colorized) and apo MvcA (gray) (panel G). The rotation of the Insertion domains from their position in the apo protein to their position in ternary complex is indicated by an arrow.

To investigate whether the opposite enzymatic activities are the consequence of divergent catalytic reactions, we searched for potential structural differences in the catalytic triads of MavC and MvcA. The two proteins utilize an identical Cys‐His‐Glu catalytic triad in which the catalytic cysteine (Cys74 in MavC and Cys83 in MvcA) is essential both for the formation and the cleavage of the isopeptide bond between Lys92 of UBE2N and Gln40 of Ub.^[11c, 12]^ In a comparable scenario, SdeA and DupA utilize an identical set of catalytic residues, but conformational differences between these residues give rise to opposite activities in phosphoribosyl ubiquitination.^[^ ^16^ ^]^ As inferred by comparison of the apo MavC and apo MvcA structures, the catalytic triads (Cys, His, Glu) of MavC and MvcA assume an identical conformation in the inactive state (**Figure** **7**A). Interestingly, comparison of MavC–UBE2N–Ub and MvcA–UBE2N–Ub ternary complexes showed that all three residues in the catalytic triads are in similarly close alignment, and the differences in positioning of the isopeptide bond between Lys92 of UBE2N and Gln40 of Ub are minute (Figure 7B). We therefore reasoned that transglutamination and deubiquitination mediated by MavC/MvcA are in fact forward and backward reactions of the same reversible catalytic reaction and that the preference for the direction in which the reaction proceeds is governed by other factors such as substrate binding affinity and stability of the substrate/product bound state. These observations and the fact that the Insertion domain and Tail domain undergo rotational movement during formation of the MavC/MvcA ternary complex prompted us to divert our attention to these two domains in further analysis.

While analyzing the Insertion domains of MavC and MvcA, we noticed that their negatively charged regions vary considerably in terms of negatively charged amino acid composition (Figure 7C). Specifically, the negatively charged region of the MavC Insertion domain contains seven negatively charged amino acids, whereas the corresponding region in MvcA contains only five, indicating that the net electric charge of the negatively charged region in MavC is more negative than the one in MvcA (Figure 7D). In both ternary complexes, the Insertion domain utilizes the negatively charged region to interact with the positively charged region of UBE2N through electrostatic forces (Figure 7E). Moreover, the conformational changes observed between the two ternary complexes and their apo form structures imply that the Insertion domains of both proteins bind UBE2N via counterclockwise rotation (Figure 7F,G). Thus, the interaction forces and mechanisms for UBE2N by MavC and MvcA are highly similar, but the binding affinity between UBE2N and MvcA is likely to be lower than that between UBE2N and MavC because of lower net negative charge.

**Figure 8:**
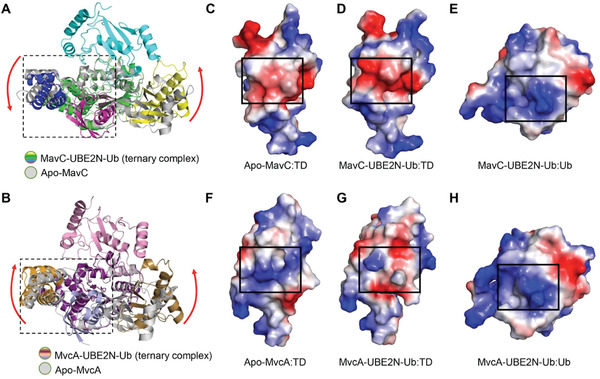
Structural comparison and analysis of Tail domains and catalytic triads of MavC and MvcA. A,B) Superimposition of MavC/MvcA–UBE2N–Ub (colorized) and apo MavC/MvcA (gray). The rotations of the Insertion domain and Tail domain from their positions in the apo MavC/MvcA to their position in the ternary complex are indicated by red arrows. C–E) Electrostatic surfaces of apo MavC Tail domain, MavC–UBE2N–Ub Tail domain and Ub. F–H) Electrostatic surfaces of apo MvcA Tail domain, MvcA–UBE2N–Ub Tail domain and Ub.

As one of the substrates, Ub mainly interacts with the Tail domain of the MavC and MvcA. Intriguingly, comparison of the MavC and MvcA ternary complexes to their apo forms shows that the Tail domain of MavC rotates counterclockwise and the Tail domain of MvcA rotates clockwise (**Figure** **8**A,B). Since the surface of Ub that interacts with the Tail domains of MavC and McvA is positively charged, the counterclockwise rotation of MavC Tail domain increases local negative potential, which likely results in tighter binding with Ub (Figure 8C,E). On the contrary, the clockwise rotation of MvcA Tail domain creates an obvious positive bulge, which probably facilitates uncoupling of Ub from UBE2N through electrostatic repulsion (Figure 8F–H).

**Figure 9:**
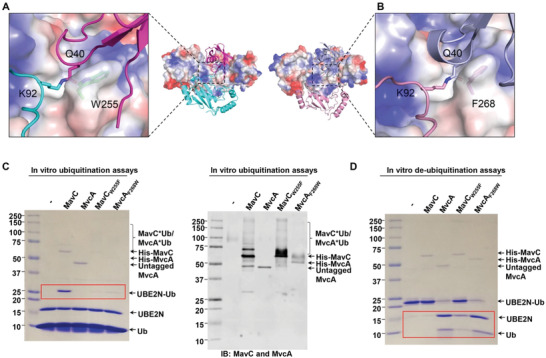
Trp255 in MavC and Phe268 in MvcA are critical for the outcome of their catalytic activity. A,B) MavC and MvcA from their ternary complex are shown as electrostatic surfaces. The isopeptide bond (cyan and magenta) in MavC–UBE2N–Ub and isopeptide bond (light blue and pink) in MavC–UBE2N–Ub are shown as sticks. Trp255 of MavC and Phe268 of MvcA in the active site are shown as ticks and labeled on the outer side of the electrostatic surface. C) The effects of MavC_W255F_ and MvcA_F268w_ on MavC‐mediated ubiquitination and self‐ubiquitination reactions. Ubiquitination reactions containing MavC, MvcA, MavC_W255F_ and MvcA_F268w_ were resolved by SDS‐PAGE and visualized by Coomassie staining (left panel) or immunoblotted with MavC and MvcA‐specific antibodies (right panel). D) The effects of MavC_W255F_ and MvcA_F268W_ on deubiquitination reaction. Proteins in deubiquitination reactions containing MavC, MvcA, MavC_W255F_ and MvcA_F268W_ were separated by SDS‐PAGE and visualized by Coomassie staining. Experiments in panel (C) and (D) were repeated at least three times and similar results were obtained.

Another difference between MavC and MvcA is a residue with its side chain adjacent to the Gln40 of Ub, i.e., Trp255 of MavC and the corresponding residue Phe268 in MvcA (Figures 9A,B and 1B, and Figure S4, Supporting Information). Considering the proximity of these two residues to the UBE2N_Lys92_–Ub_Gln40_ isopeptide bond and their distinct properties, we wondered whether they play any role in dictating the outcome of the reactions catalyzed by MavC and MvcA. To investigate this, we constructed mutants MavC_W255F_ and MvcA_F268W_ and tested their function in ubiquitination and deubiquitination of UBE2N. Surprisingly, MavC_W255F_ exhibited lower efficiency in UBE2N ubiquitination than MavC_WT_ but has gained the ability to cleave the isopeptide bond between UBE2N and Ub (**Figure** **9**C,D). Similarly, MvcA_F268W_ has been equipped with the ability to ubiquitinate UBE2N, but it also retained the ability to deubiquitinate UBE2N–Ub at levels indistinguishable from that of MvcA (Figure 9C,D). Intriguingly, self‐ubiquitination, which is a property specific for MavC but not MvcA, was detected in reaction with MvcA_F268W_ and was further enhanced for MavC_W255F_ (Figure 9C, right panel). These findings confirm that Trp255 in MavC and its counterpart Phe268 in MvcA are one of the key residues that define the propensity of MavC for UBE2N ubiquitination and self‐ubiquitination, and MvcA for UBE2N–Ub deubiquitination, respectively.

**Figure 10:**
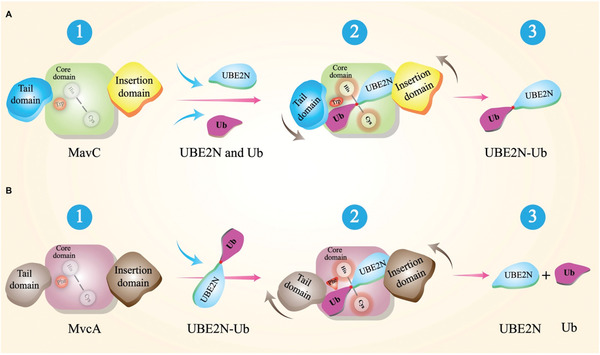
Models for mechanisms underlying MavC transglutamination and MvcA deubiquitination activities. A,B) UBE2N–Ub is produced by MavC via transglutamination (A, step 1 to step 2) and UBE2N–Ub is hydrolyzed to UBE2N and Ub by MvcA deubiquitination (B, step 1 to step 2) with rotation of their Insertion domains and Tail domains in directions indicated by gray arrows (step 2). Products release and the completion of the catalytic reactions (step 3).

In conclusion, based on the extensive structural analyses and experimental results, we propose a model for the molecular basis for the opposite enzymatic activities of MavC and MvcA in their regulation of UBE2N ubiquitination (**Figure** **10**). The initial substrate engagement of MavC/MvcA involves binding of UBE2N and Ub/crosslinked UBE2N–Ub through interactions that induce rotation of the Insertion domain and the Tail domain, which in turn positions UBE2N and Ub/UBE2N–Ub in the active site of the respective protein. In MavC, the rotation of the Insertion domain and the Tail domain results in more stable binding between the UBE2N and Ub and MavC, which along with Trp255 creates conditions favorable for transglutamination (Figure 10A). Conversely, the repulsive forces between Ub and Tail domain caused by its clockwise rotation and lower binding affinity of the Insertion domain for UBE2N work in synergy with Phe268 in MvcA to promote the dissociation of Ub and UBE2N from the enzyme, thus leading to deubiquitination (Figure 10B).

## 3 Discussion

MavC is a transglutaminase that catalyzes covalent crosslinking of Gln40 of ubiquitin to either Lys94 or Lys92 of UBE2N (Figure S1C, Supporting Information), and it also possess deamidase activity that targets Ub at Gln40 in the absence of UBE2N (Figure S1D, Supporting Information).^[^ ^14^ ^]^ The effect of MavC is counteracted by MvcA, which is a close homolog of MavC that functions as a deubiquitinase against UBE2N–Ub (Figure 1B).^[^ ^12^ ^]^ Like MavC, MvcA also exhibits ubiquitin deamidase activity in a manner similar to bacterial deamidases such as Cif and CHBP that deamidate Gln40 of Ub and Ub/NED88, respectively.^[^ ^15^ ^]^ Structural comparison of these four enzymes reveals that the structures of CHBP and Cif align strikingly well with the Core and Tail domains of MavC and MvcA (Figure 1C). However, the Insertion domain appears to be characteristic of MavC and MvcA as its structural equivalent is absent in CHBP and Cif (Figure 1). Therefore, we reason that the Core and Tail domains of MavC and MvcA are sufficient for their ubiquitin deamidation activity. Indeed, deletion of the Insertion domain abolishes the transglutamination activity of MavC while it largely retains its deamidation activity (Figure 2).

That being said, what is the functional significance of the Insertion domain? CHBP is known to target multiple signaling pathways with its ubiquitin deamidation activity via blocking free ubiquitin chain synthesis by different E3–E2 pairs, leading to the inhibition of ubiquitination of RhoA mediated by a Cullin‐based E3 complex, and subsequently cell cycle arrest.^[^ ^17^ ^]^ In contrast, the scope of activity for MvcA and MavC appears narrower as they specifically regulate ubiquitination in UBE2N‐related pathways such as NF‐κB signaling.^[^ ^18^ ^]^ Previously reported structure of the MvcA–UBE2N–Ub ternary complex reveals that the Insertion domain is involved in recognition of the UBE2N–Ub substrate.^[^ ^12^ ^]^ In line with that, our study shows that the Insertion domain of MavC is also involved in the recognition of UBE2N.^[11c]^ Hence, the Insertion domain enables MvcA and MavC to act specifically on UBE2N, thereby making the regulation of host ubiquitination signaling by MavC and MvcA more directed and precise.

Catalytic domains that mediate chemically opposite reactions in highly homologous proteins are not unprecedented: the PDE domains of DupA/B and SidE enzymes are another example. PDE domains of SidE enzymes have moderate binding affinity for Ub and catalyze PR ubiquitination, whereas PDE domains of DupA/B bind strongly to Ub and mediate the removal of PR‐Ub. The formation of stable enzyme–substrate complexes is required to mediate cleavage reaction, while the ligation reaction requires moderate binding affinity to substrates, probably allowing efficient release of products.^[^ ^16^ ^]^ MavC and MvcA are highly similar proteins with ≈50% sequence identity and identical catalytic triad (Figure 1B), yet MavC primarily catalyzes the formation of an isopeptide bond between UBE2N_Lys92_ and Ub_Gln40_, whereas MvcA is responsible for breaking of the same isopeptide bond. The structure of MavC ternary complex shows that the negatively charged surface of Tail domain attracts the positively charged surface of Ub, resulting in stable binding between MavC and Ub. Concurrently, the counterclockwise rotation of the Ub‐bound Tail domain draws Ub closer to UBE2N and the active center to facilitate isopeptide bond formation. In MvcA, however, the surface charge of Tail domain is repulsive to the Ub and lower binding affinity for UBE2N favors the dissociation of Ub from MvcA, which in turn facilitates the isopeptide bond cleavage and the separation of Ub and UBE2N from MvcA. Besides, Trp255 in MavC and Phe268 in MvcA also play crucial role in determining the direction in which the enzymatic reaction proceeds. Although the position and direction of these two residues is roughly equal in the ternary complexes, comparison between apo and ternary structures of MavC and MvcA reveals that Trp255 and Phe268 in fact make opposite movements during ternary complex formation to reach that position: Trp255 slightly shifts backward in MavC, whereas Phe268 makes a relatively larger shift toward Gln40 of Ub in MvcA. Taking into account the vicinity of the Tail domain and the direction of these positional shifts, we speculate that they are driven by counterclockwise rotation/clockwise rotation of the Tail domains of respective proteins. Together with the distinct properties of tryptophan and phenylalanine, the differences in positional shifts of Trp255 and Phe268 can provide a more comprehensive explanation for the opposite enzymatic activities of MavC and MvcA. With its high hydrophobicity and “pushing motion,” Phe268 is likely to exert steric strain on Gln40 of Ub, which may in turn destabilize the isopeptide bond by creating torsional stress; along with electrostatic repulsion between the Tail domain and Ub, this would increase the overall entropy of the MvcA–UBE2N–Ub complex, resulting in the cleavage of the isopeptide bond and dissociation of UBE2N and Ub from MvcA. In contrast, the amphipathicity of Trp255 and its “withdrawing motion” could serve to accommodate Gln40 of Ub and stably hold it in place without steric strain so that the synthesis of the UBE2N_Lys92_–Ub_Gln40_ isopeptide bond can be carried out by the catalytic center in MavC. Thus, given their overall structural similarity and equivalent chemical nature of the ubiquitin ligation by transglutamination and deubiquitination activities, the fact that MavC and MvcA are licensed with opposite enzymatic activities is due to differences in substrate binding preference and stability of the substrate/product‐bound intermediates. We propose that the formation of stable enzyme–substrate complexes is required for the isopeptide bond synthesis reaction, whereas weak and/or repulsive interactions between enzyme and product favor cleavage reactions.

For the transglutaminase activity of MavC, the Insertion domain enables the specific binding of MavC to UBE2N, whereas the Tail domain maintains the binding with Ub, and the Core domain serves as scaffold to stabilize UBE2N_Lys92_ and Ub_Gln40_ in the catalytic center. These three domains coordinate to complete the catalytic reaction. The shape complementarity and architecture, rather than specific individual interactions, is crucial for isopeptide bond formation between UBE2N and Ub. For the deamidase activity of MavC, the Insertion domain is dispensable. In the catalyzing process of MavC, Cys74 first attacks Gln40 in ubiquitin to form a thioester intermediate (Figure S7, Supporting Information). When UBE2N is present, the acylated MavC reacts with the amine donor from the ε‐lysine in UBE2N to form an intermolecular isopeptide bond. In the absence of UBE2N, the acylated MavC is further attacked by a nucleophilic water to produce Ub_Glu40_. MvcA can deubiquitinate the UBE2N–Ub product from MavC transglutaminase activity (Figure S7, Supporting Information).^[11c, 12]^

As a key checkpoint for activation of the NF‐κB pathway, UBE2N appears to be a common target of such cellular subversion as bacterial infection; it can be regulated by diverse mechanisms such as deamidation and ISGylation.^[^ ^3^ ^,^ ^19^ ^]^ MavC and MvcA regulate the activity of UBE2N in spatial and temporal manner via their opposite enzymatic activities. Crosslinking of UBE2N and Ub catalyzed by MavC leads to the inhibition of the UBE2N‐mediated NF‐κB activation, which is essential in the early infection, whereas the deubiquitinase activity of MvcA restores the activity of UBE2N to allow the recovery of host signaling pathways in the later phases of infection when the bacteria have already successfully evaded host detection. Such mechanisms may allow fewer disruptions to the pathogen or promote symbiotic coexistence between the pathogen and their hosts under conditions of evolutionary pressure.

## 4 Experimental Section

##### Media, Bacteria Strains, Plasmid Constructions, and Cell Lines

*Legionella* strains used in this papers were derivatives of *Philadelphia* 1 strain Lp02 and were grown and maintained on charcoal‐yeast extract (CYE) plates or in AYE.^[^ ^20^ ^]^ For complementation experiments, the genes were cloned into pZL507.^[^ ^21^ ^]^ *Escherichia coli* strains XL1‐Blue and BL21(DE3) grown in Luria broth (LB) were used for expression and purification of all the recombinant proteins. Genes for protein purification were cloned into pQE30 (QIAGEN). Raw 264.7 and U937 cells were cultured in RPIM1640 medium in the presence of 10% FBS. When necessary, U937 cells were differentiated into macrophages by phorbol 12‐myristate 13‐acetate as described earlier.^[^ ^22^ ^]^ All cell lines were directly purchased from ATCC.

##### Purification of Proteins for Biochemical Experiments

For protein production, 10 mL overnight cultures were transferred to 200 mL LB medium in the presence of 100 µg ampicillin and grown to OD_600 nm_ of 0.6–0.8. The cultures were then incubated at 18 °C for 16–18 h after adding isopropyl β‐d‐1‐thiogalactopyranoside (IPTG) to a final concentration of 0.2 × 10^−3^ m. Bacterial cells were harvested at 12 000 × *g* by spinning and lysed by sonication. The soluble lysates were cleared by spinning at 12 000 × *g* twice at 4 °C for 20 min. His‐tagged proteins were purified with Ni^2+^‐NTA beads (Qiagen) and eluted with 300 × 10^−3^ m imidazole in PBS buffer. Purified proteins were dialyzed with buffer containing 50 × 10^−3^ m Tris–HCl (pH7.5), 150 × 10^−3^ m NaCl, 5% glycerol, and 1 × 10^−3^ m DTT.

##### Purification of Proteins for Structural Study

The gene of MavC (full length) was PCR amplified from *L. pneumophila* genomic DNA and inserted into pGEX‐6p‐1. The gene sequences of MavC_C74A_ (7‐384 AA, Cys74 residue mutated to Ala) and UBE2N_K94A_ (full length) were also inserted into pGEX‐6p‐1. The gene sequence of Ub (1‐76 AA) was inserted into pQE30. These plasmids were transformed into *E. coli* BL21(DE3) cells. The cells were grown in LB medium at 37 °C with constant shaking at 220 rpm until the cell concentration reached OD_600_ of 0.8, after which the recombinant protein expression was induced by the addition of IPTG to a final concentration of 0.3 × 10^−3^ m. The recombinant proteins were expressed at 18 °C for 16 h. The cells were pelleted by centrifugation (5000 × *g*, 15 min) and subsequently resuspended in the cold lysis buffer (50 × 10^−3^ m Tris–HCl pH 7.5, 150 × 10^−3^ m NaCl). Following lysis by ultrasonication, the lysates were centrifuged at 17 000 rpm for 30 min at 4 °C. The proteins with glutathione S‐transferase (GST) tag or His tag were purified by affinity chromatography (glutathione agarose resin and Ni^2+^ resin, respectively). The GST tag was removed by the PreScission protease (PPase). The tag‐less protein was then purified by size‐exclusion chromatography (SEC) using a Superdex 200 increase column (GE Healthcare) equilibrated with a buffer containing 25 × 10^−3^ m 4‐(2‐hydroxyethyl)‐1‐piperazineethanesulfonic acid (HEPES), pH 7.5, 150 × 10^−3^ m NaCl, and 2 × 10^−3^ m DTT.

To prepare the MavC_C74A_–UBE2N_K94A_ complex, UBE2N_K94A_ was incubated with MavC_C74A_ at 2:1 molar ratio at 4 °C for 1 h. To crosslink UBE2N_K94A_ and Ub, wild‐type MavC was incubated with UBE2N_K94A_ and Ub at a molar ratio of 1:120:180 in a reaction buffer containing 25 × 10^−3^ m HEPES, pH 7.5, 150 × 10^−3^ m NaCl, 2 × 10^−3^ m Dithiothreitol (DTT), and 10 × 10^−3^ m Mg^2+^ at 25 °C for 5 min. The UBE2N_K94A_–Ub was separated from MavC and excess UBE2N by Ni^2+^‐NTA beads at 4 °C and then purified by gel filtration to remove the excess Ub.^[^ ^12^ ^]^ The UBE2N_K94A_–Ub was collected and incubated with MavC_C74A_ at 1:2 molar ratio at 4 °C for 1 h. Finally, the MavC_C74A_–UBE2N–Ub complex was separated by gel filtration, pooled, and concentrated to 14.9 mg mL^−1^ for the use in crystallization screen.

##### Crystallization, Data Collection, and Structural Determination

Crystallization screens were performed using the sitting drop vapor diffusion method at 16 °C, with drops containing 0.5 µL of the protein solution mixed with 0.5 µL of reservoir solution. Diffraction‐quality MavC_C74A_–UBE2N_K94A_–Ub crystals were obtained in 0.2 m sodium malonate pH 6.0, 20% w/v PEG 3350, whereas MavC_C74A_–UBE2N_K94A_ crystals were obtained in 0.2 m sodium malonate pH 7.0, 20% w/v PEG 3350. The crystals were harvested and flash‐frozen in liquid nitrogen with 20% glycerol as cryoprotectant. Complete X‐ray diffraction datasets were collected at BL‐17U1 beamline of Shanghai Synchrotron Radiation Facility (SSRF). Diffraction images were processed with the HKL‐2000 program.^[^ ^23^ ^]^ Structures of the binary and ternary complex were solved by molecular replacement (MR) using Phaser‐MR (MavC: 5TSC, UBE2N: 1JAT, Ub: 4HCN).^[^ ^14^, ^24^ ^]^ Model building and crystallographic refinement were carried out in Coot and PHENIX.^[^ ^25^ ^]^ The quality of the final model was validated with MolProbity.^[^ ^26^ ^]^ The interactions were analyzed with PyMol (http://www.pymol.org/) and PDBsum. The figures were generated in PyMol. Detailed data collection and refinement statistics are listed in Table 1.

##### MavC‐Induced Ubiquitination and Deamidation Assays

For MavC‐mediated ubiquitination of UBE2N, 5 µg Ub, 0.5 µg UBE2N or its mutant derivatives and 0.05 µg MavC or its mutant derivatives were incubated for 8 or 30 min at 37 °C in 25 µL reactions systems containing 50 × 10^−3^ m Tris–HCl (pH7.5), 5 × 10^−3^ m Mn^2+^, and 1 × 10^−3^ m DTT. Reaction products were resolved by sodium dodecyl sulphate‐polyacrylamide gel electrophoresis (SDS‐PAGE), protein bands were visualized by Coomassie brilliant blue staining and the amount of protein in bands was quantified by densitometry.

For MavC‐mediated deamidation of Ub, 10 µg Ub and 1 µg MavC, or its mutant derivatives were incubated for 1 h at 37 °C in 25 µL reactions containing 50 × 10^−3^ m Tris–HCl (pH 8.8). Reaction mixtures were then mixed with 5× native gel loading buffer and resolved by native PAGE followed by Coomassie brilliant blue staining.

##### MavC‐Mediated Ubiquitination of UBE2N during *L. pneumophila* Infection

For *L. pneumophila* infection experiments, all *Legionella* strains including complementation strains were grown overnight in AYE medium to post‐exponential phase (OD_600nm_ = 3.2–3.8) and were induced with 0.2 × 10^−3^ m IPTG for 3 h at 37 °C before infection. Raw264.7 or U937 cells were infected with *L. pneumophila* strains at a multiplicity of infection (MOI) of 10 for 2 h. Cells washed with phosphate‐buffered saline (PBS) three times were collected and lysed with 0.2% saponin on ice for 30 min. The cell lysates were resolved by SDS‐PAGE and probed with MavC‐specific antibody to check the translocation of MavC and its mutants, and modified UBE2N was probed with UBE2N‐specific antibody.

## Conflict of Interest

The authors declare no conflict of interest.

## Author Contributions

H.G., J.F., T.Y., and Z.‐X.W. contributed equally to this work. S.O. and Z.‐Q.L. conceived the project. H.G., T.Y., and Y.H. crystalized the complexes and determined the structures; Y.L. made the movie; J.F. and N.G. constructed and analyzed the mutants; H.G., Z.‐X.W., Z.‐Q.L., J.F., and S.O. analyzed the data. H.G., J.F., Z.‐X.W., V.P., Z.‐Q.L., and S.O. wrote the manuscript.

## Supporting information

Supporting Information

Supplemental Movie 1
