# Supplementary material for: Molecular Basis of Ubiquitination Catalyzed by the Bacterial Transglutaminase MavC
Source: Adv Sci (Weinh). 2020 Apr 30;7(12):2000871. doi: 10.1002/advs.202000871 (PMC7312448; doi:10.1002/advs.202000871)
Supplement: Supplementary file 1 — Supporting Information [file ADVS-7-2000871-s002.pdf]

Supporting Information

Molecular basis of ubiquitination catalyzed by the bacterial transglutaminase MavC

Hongxin Guan, Jiaqi Fu, Ting Yu, Zhao-Xi Wang, Ninghai Gan, Yini Huang, Vanja Perčulija, Yu Li, Zhao-Qing Luo and Songying Ouyang**

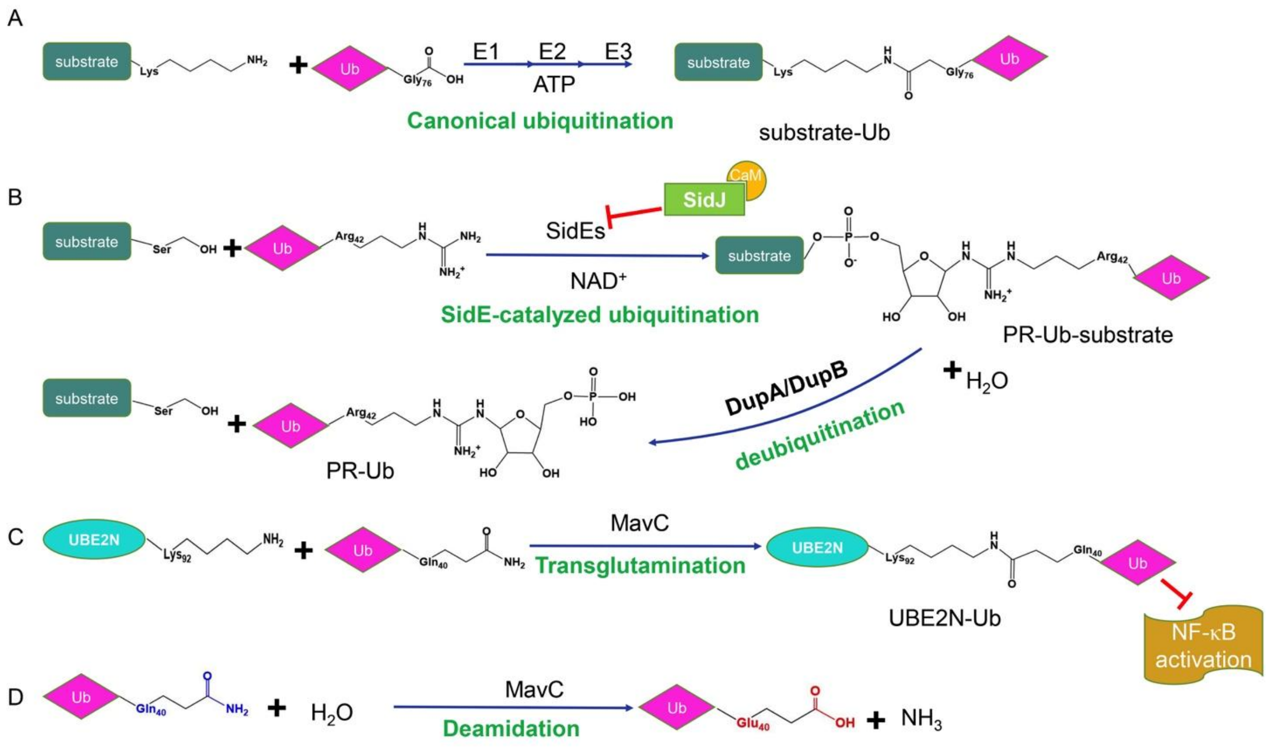

Figure S1. Mechanisms of ubiquitination and deubiquitination. **A,** General scheme of canonical ubiquitination. **B,** SidE-catalyzed ubiquitination that is negatively regulated by SidJ/CaM and DupA/DupB-catalyzed deubiquitination. **C,** MavC-catalyzed transglutamination. The product UBE2N-Ub suppresses the activation of NF-κB. **D,** MavC-catalyzed deamidation of Ub in solution.

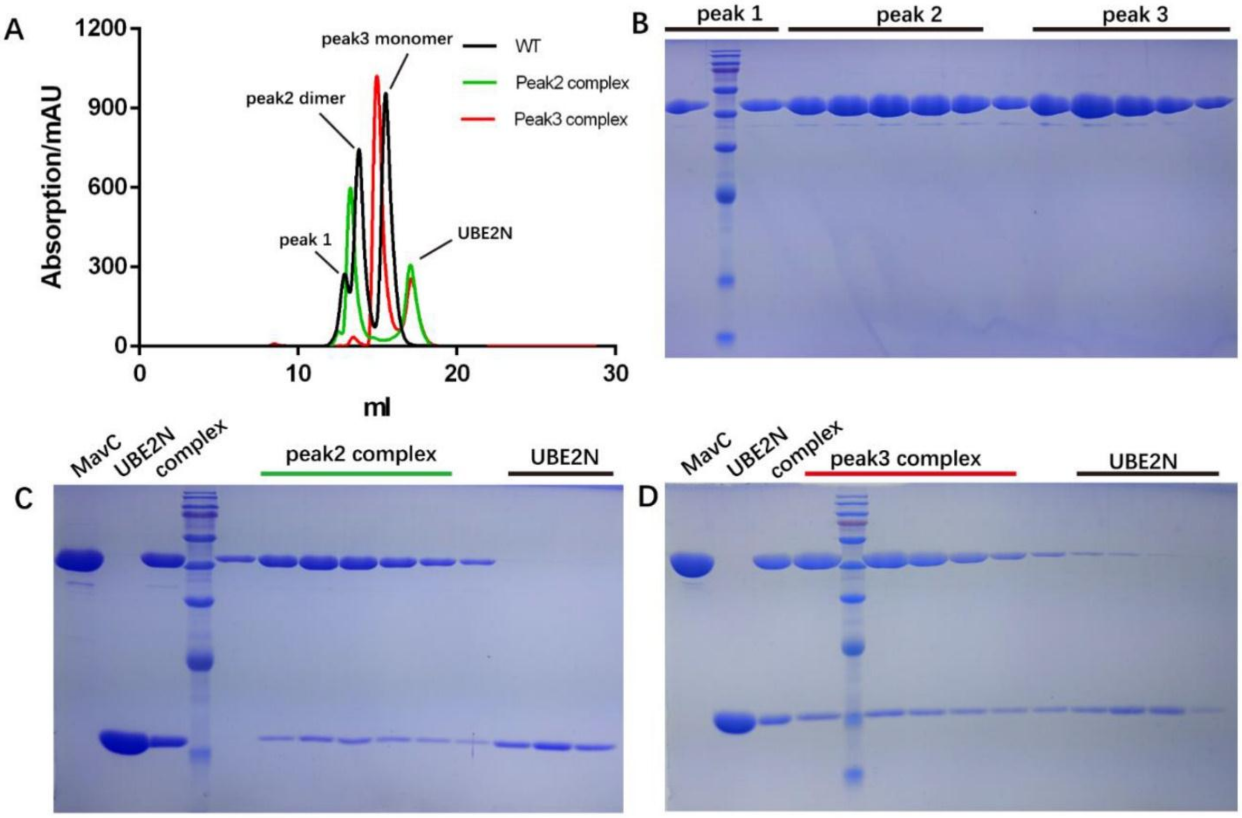

Figure S2. Two variants of MavC-UBE2N complexes used for crystallization. **A,** MavC eluted from peak 2 and peak 3 in gel filtration was incubated with UBE2N at a ratio of 1:2 and purified by size-exclusion chromatography using a Superdex200 increase column. The figure compares retention volumes of MavC (black), MavC (peak2) -UBE2N complex (green) and MavC (peak3) -UBE2N (red) complex. **B-D,** The elution peaks of MavC and MavC-UBE2N complexes were examined by SDS-PAGE. The word “complex” in the third lane 3 of gel images (C) and (D) is used to denote complex samples before gel filtration.

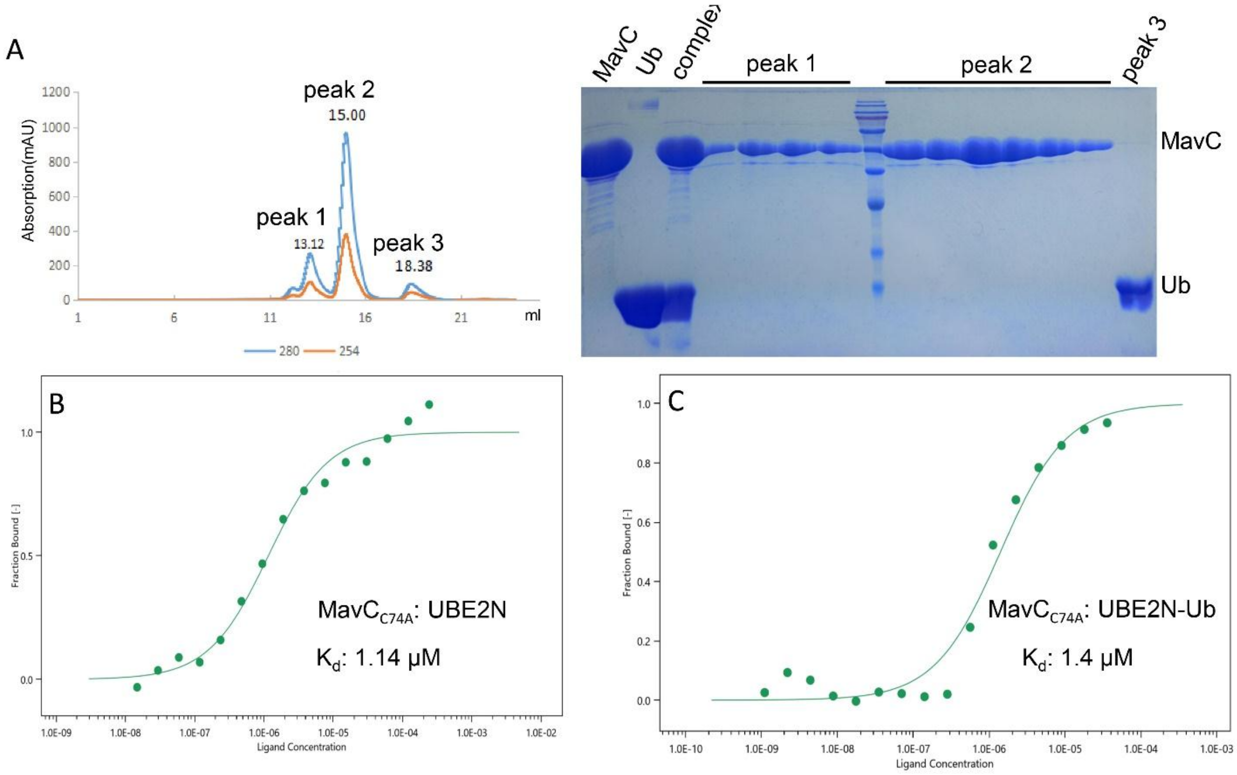

Figure S3. Interactions of MavC and Ub, MavC and UBE2N, and MavC and UBE2N-Ub in vitro. **A,** Purified MavC was incubated with Ub at a ratio of 2:1 and purified by size-exclusion chromatography using a Superdex200 Increase column. Peak 1 (dimeric MavC), peak 2 (monomeric MavC) and peak3 (Ub) were examined by SDS-PAGE. The word "complex" in the third lane 3 of gel images is used to denote complex samples before gel filtration. **B-C,** The interactions between $MavC_{C74A}$ and UBE2N (panel **B**), and $MavC_{C74A}$ and UBE2N-Ub (panel **C**) were measured by microscale thermophoresis (MST).

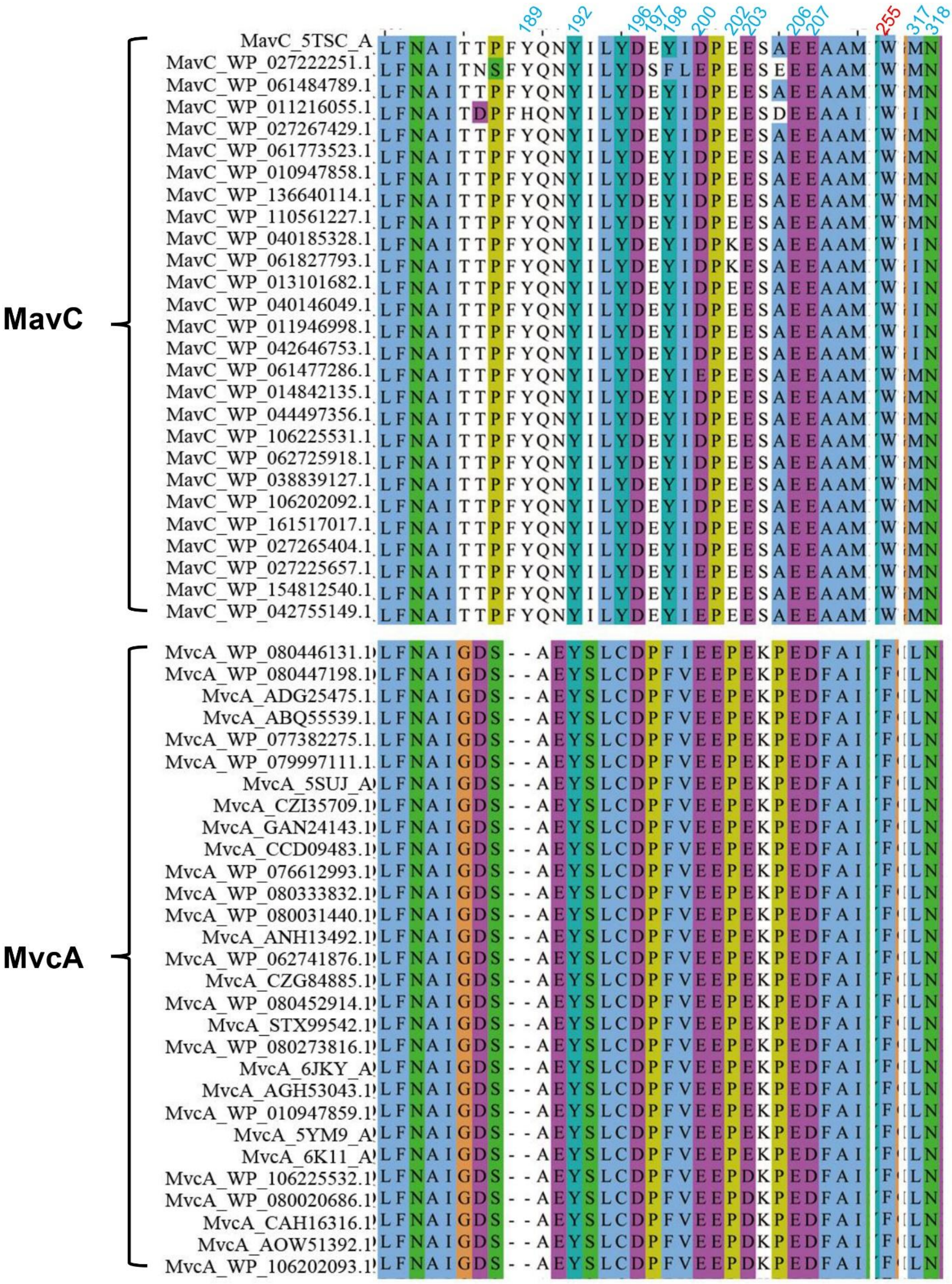

Figure S4. Multiple sequence alignment of MavC and MvcA sequences. MavC and MvcA sequences alignments carried out by using MUSCLE v3.8.31 and visualized with Jalview v2.10.3.

Residues involved in electrostatic interactions between the negative surface of MavC and positive surface of UBE2N are labeled with cyan. Trp255 of MavC in the active site is labeled with red.

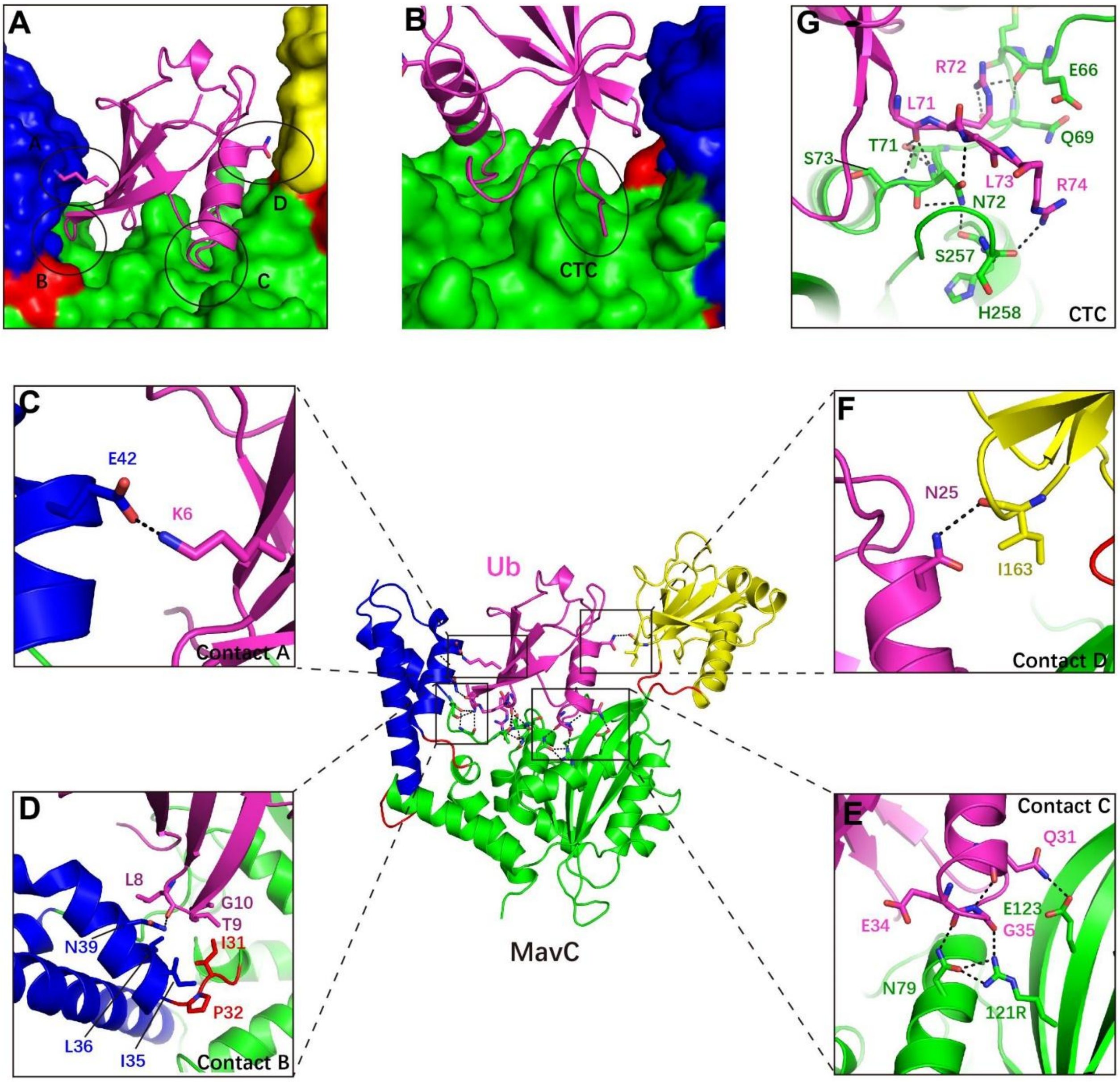

Figure S5. Interactions between MavC and Ub in the MavC-UBE2N-Ub complex. **A and B,** The five contacting interfaces between MavC (green-yellow-blue surface) and Ub (magenta cartoon) are indicated by circles. **C-G,** Detailed views of contact A (panel C), contact B (panel D), contact C (panel E), contact D (panel F), and the C-terminal contact (CTC) (panel G). Key residues involved

in interactions between MavC (green-yellow-blue) and Ub (magenta cartoon) are shown as sticks. Hydrogen bonds are indicated by black dashed lines.

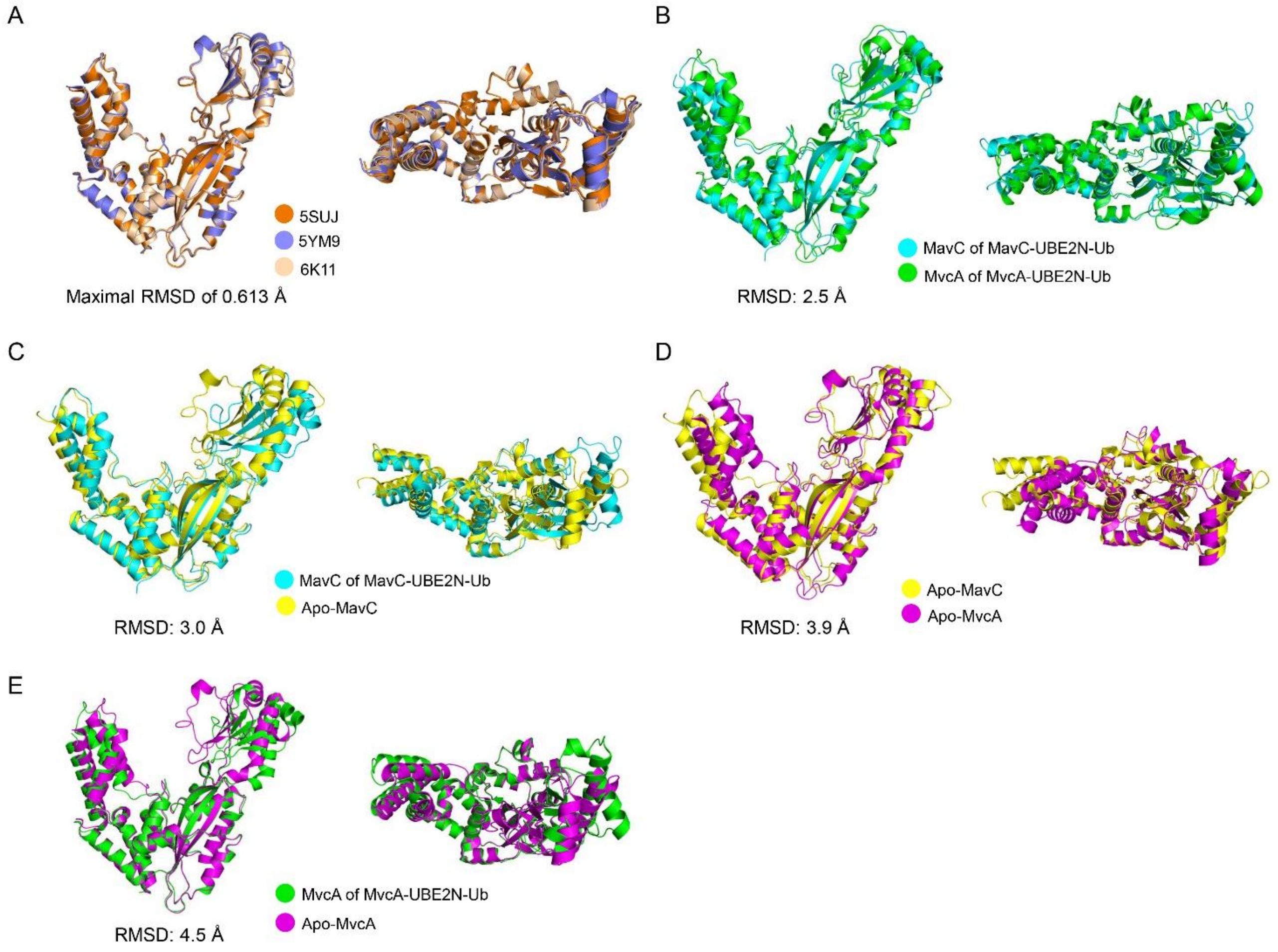

Figure S6. Structural alignments of MavC and MvcA in apo form and ternary complex. **A,** Structural alignment of three available apo MvcA structures (PDB ID: 5SUJ, 5YM9, 6K11) with a maximal RMSD of 0.613 Å. **B,** Structural alignment of the ternary complex of MavC-UBE2N-Ub (cyan) and MvcA-UBE2N-Ub (green) with an RMSD of 2.5 Å. **C,** Structural alignment of the MavC-UBE2N-Ub ternary complex (cyan) and apo MavC (yellow) with an RMSD of 3.0 Å. **D,** Structural alignment of apo MavC (yellow) and apo MvcA (magenta) with an RMSD of 3.9 Å. **E,** Structural alignment of the MvcA-UBE2N-Ub ternary complex (green) and apo MvcA (magenta)

with an RMSD of 4.5 Å.

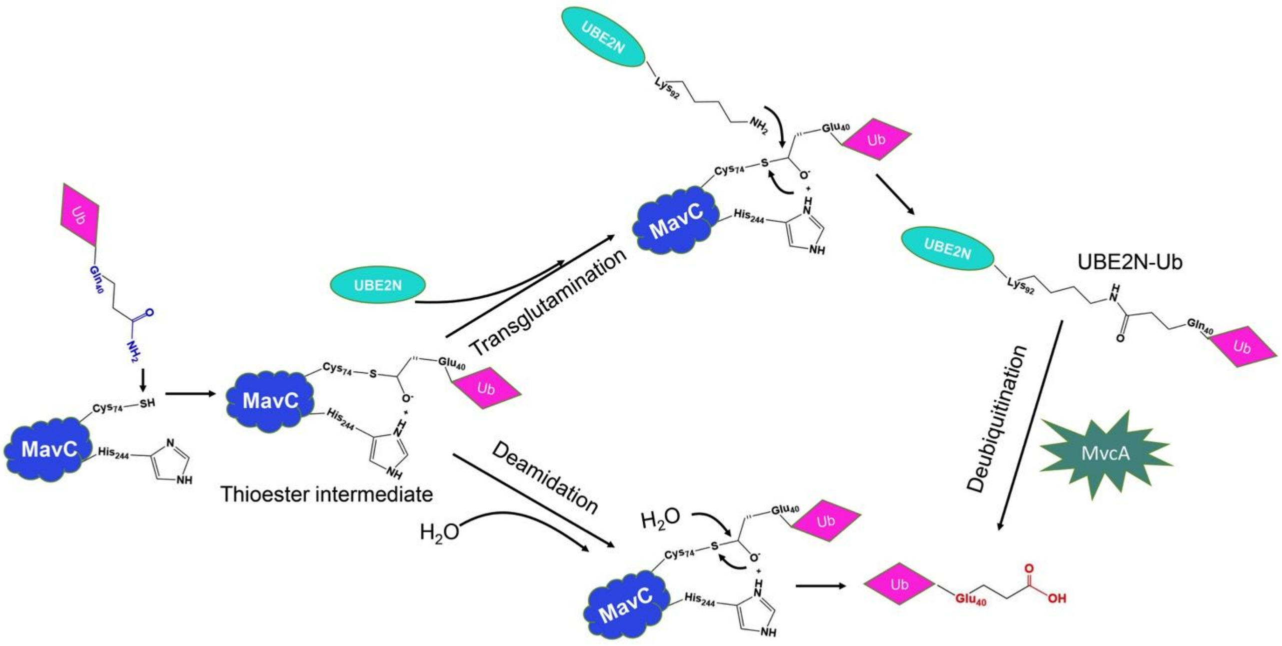

Figure S7. Proposed mechanisms of transglutamination and deamidation reaction catalyzed by MavC and deubiquitination reaction catalyzed by MvcA. In the first step, Cys74 of MavC attacks Gln40 of Ub to generate a thioester intermediate. This intermediate reacts with Lys92 on UBE2N to form UBE2N-Ub or with H_2O to form Glu40 in the absence of UBE2N. The ispoeptide bond in UBE2N-Ub complex catalyzed by MavC can be cleaved by MvcA.

Movie S1. Conformational changes in MavC and MvcA during their transition from apo form to ternary complex. MavC exhibits concave architecure in which the Insertion domain (yellow) and Tail domain (blue) flank the Core domain (green). The transglutaminase catalytic site is located in the Core domain, at the bottom of concavity of MavC. Upon binding to UBE2N and Ub, both Insertion domain and Tail domain undergo anticlockwise rotation that facilitates interactions with UBE2N and Ub, respectively. Comparison of MavC and MvcA in their apo form and ternary complexes reveals that the Insertion domain and Tail domain also rotate during binding with

UBE2N-Ub, but the Tail domain of MvcA rotates clockwise instead of anticlockwise.
